# Subdural spread of injected local anesthetic in a selective transforaminal cervical nerve root block: a case report

**DOI:** 10.1186/1752-1947-6-142

**Published:** 2012-06-01

**Authors:** Katsuhiro Tofuku, Hiroaki Koga, Setsuro Komiya

**Affiliations:** 1 Department of Orthopedic Surgery, Imakiire General Hospital, Kagoshima, Japan; 2 Department of Orthopedic Surgery, Kagoshima Graduate School of Medical and Dental Sciences, Kagoshima, Japan

## Abstract

**Introduction:**

Although uncommon, selective cervical nerve root blocks can have serious complications. The most serious complications that have been reported include cerebral infarction, spinal cord infarction, transient quadriplegia and death.

**Case presentation:**

A 40-year-old Japanese woman with a history of severe right-sided cervical radicular pain was scheduled to undergo a right-sided C6 selective cervical nerve root block using a transforaminal approach under fluoroscopic guidance. An anterior oblique view of the C5-C6 intervertebral foramen was obtained, and a 23-gauge spinal needle, connected to the normal extension tube with a syringe filled with contrast medium, was introduced into the posterior-caudal aspect of the C5-C6 intervertebral foramen on the right side. In the anteroposterior view, the placement of the needle was considered satisfactory when it was placed no more medial than halfway across the width of the articular pillar. Although the spread of the contrast medium along the C6 nerve root was observed with right-sided C6 radiculography, the subdural flow of the contrast medium was not observed with real-time fluoroscopy. The extension tube used for the radiculography was removed from the spinal needle and a normal extension tube with a syringe filled with lidocaine connected in its place. We performed a negative aspiration test and then injected 1.5 mL of 1.0% lidocaine slowly around the C6 nerve root. Immediately after the injection of the local anesthetic, our patient developed acute flaccid paralysis, complained of breathing difficulties and became unresponsive; her respiratory pattern was uncoordinated. After 20 minutes, she regained consciousness and became alert, and her muscle strength in all four limbs returned to normal without any sensory deficits after receiving emergent cardiorespiratory support.

**Conclusions:**

We believe that confirming maintenance of the appropriate needle position in the anteroposterior view by injecting local anesthetic is important for preventing central needle movement. Because the potential risk of serious complications cannot be completely eliminated during the use of any established selective cervical nerve root block procedure, preparation for an emergency airway, ventilation and cardiovascular support is indispensable in cases of high spinal cord anesthesia.

## Introduction

Selective cervical nerve root block (SCNRB) is a valuable technique for the evaluation of cervical radiculopathy in patients with multilevel disease or when there is no correlation between clinical findings and radiological pathology. In addition, SCNRB is used as a therapeutic procedure in patients with cervical radiculopathy. There is consensus that image guidance techniques, including fluoroscopy, computed tomography (CT) and CT fluoroscopy, are required to safely and accurately establish an SCNRB. Meticulous attention to needle placement with image guidance is indispensable in preventing neurologic complications.

SCNRB can have serious complications, although uncommon. The most serious complications that have been reported include cerebral infarction, spinal cord infarction, transient quadriplegia and death [1-7]. SCNRB poses the potential risk of injection into the vertebral artery, radicular artery or the subdural space. We describe a case of high spinal cord anesthesia that occurred during transforaminal SCNRB due to the puncture of the epidural sac of the nerve root sleeve with subdural spread of the injected local anesthetic.

## Case presentation

A 40-year-old Japanese woman presented with severe right-sided cervical radicular pain, which had resisted medication and rehabilitation. Magnetic resonance imaging of her cervical spine revealed a cervical herniated disc on the right side at the level of the C5-C6 and C6-C7 vertebrae. She was scheduled to undergo a right-sided C6 SCNRB for diagnostic and therapeutic purposes. She had no relevant medical history. Magnetic resonance imaging revealed no abnormal findings, such as a perineural cyst or an ectatic nerve root sleeve, at the right C5-C6 intervertebral foramen. Her heart rate was 71 beats per minute, her blood pressure (BP) 110/65 mmHg and her oxygen saturation (SpO_2_) level was 100%.

The procedure was performed with our patient in the supine position. Our patient’s head was turned to the left and secured. An anterior oblique view of the C5-C6 intervertebral foramen was obtained, and a 23-gauge spinal needle, connected to the normal extension tube with a syringe filled with contrast medium, was introduced into the posterior-caudal aspect of the C5-C6 intervertebral foramen on the right side under fluoroscopic guidance. In the anteroposterior view, the placement of the needle was considered satisfactory when it was placed no more medial than halfway across the width of the articular pillar. Because careful aspiration with a 2 mL syringe did not show any blood or cerebrospinal fluid, 1.0 mL of contrast medium was injected. Although the spread of the contrast medium along the C6 nerve root was observed, subdural flow of the contrast medium was not observed very well with real-time fluoroscopy. The extension tube used for the radiculography was removed from the spinal needle and a normal extension tube with a syringe filled with lidocaine connected in its place. We performed a negative aspiration test and then injected 1.5 mL of 1.0% lidocaine slowly around the C6 nerve root. The microbore extension tubing (pigtail) was not used in the procedure.

**Figure 1:**
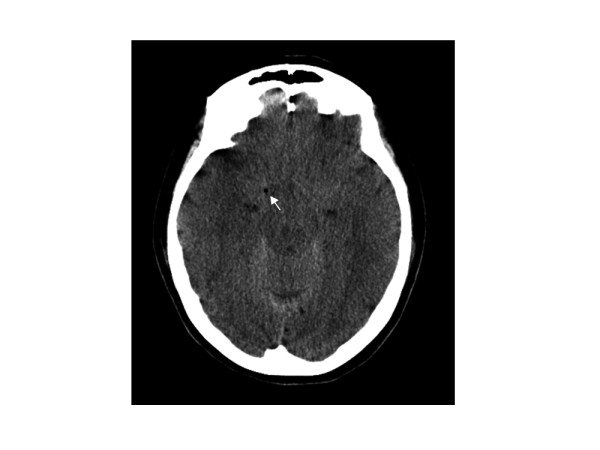
Computed tomographic scan of the brain obtained after the event revealed the presence of air gases (arrow), which suggested the infusion of subdural air while attaching an extension tube with a syringe filled with a local anesthetic.

Immediately after the injection of the local anesthetic, our patient developed acute flaccid paralysis, complained of breathing difficulties and became unresponsive. Her BP and SpO_2_ changed to 65/46 mmHg and 83%, respectively, and her respiratory pattern was uncoordinated. Her SpO_2_ improved to 98% after oxygen was supplied at 5 L/min by a Hudson mask, and her BP improved after the intravenous administration of a total of 0.5 mg of epinephrine. Thereafter, her cardiorespiratory status remained stable. A CT scan of her brain performed at this time revealed the presence of air gases, which suggested that a subdural injection of air occurred due to the clumsy method of attaching the extension tube filled with local anesthetic (Figure 1). After 20 minutes, she regained consciousness and became alert. Her muscle strength in all four limbs returned to normal without sensory deficits. Our patient was transferred to a recovery room, kept under close observation and discharged the next day as she had no persisting neurological deficits or other impairments.

## Discussion

We describe a case of high spinal cord anesthesia that occurred due to the puncture of the epidural sac of the nerve root sleeve with the subdural spread of injected local anesthetic during the transforaminal procedure. In a large series of lumbar epidural injections, Lubenow *et al*. [8] reported that the incidence of inadvertent subdural block was 0.82% and that the three most common features were an unexpectedly high sensory block, exaggerated hypotension and an unexpected motor block. We considered that the central movement of the needle tip might occur, which could result in the puncture of the epidural sac of the nerve root sleeve. We should have injected the local anesthetic whilst confirming the maintenance of the appropriate needle position - no more medial than halfway across the width of the articular pillar in the anteroposterior view. The use of microbore extension tubing (pigtail) to minimize needle manipulation while changing syringes has been recommended by several authors [5,9].

Furman *et al*. [10] reported that, among 504 transforaminal cervical epidural steroid injections, 19.4% were intravascular contrast injections; they concluded that the presence of blood in the needle hub was not a reliable predictor of intravascular needle tip placement because it was only 45.9% sensitive. Several authors have reported cases of cervical spinal cord infarction or temporary anesthesia of the spinal cord resulting from impaired perfusion of the radicular artery after injections of corticosteroids or local anesthetic in SCNRB when using a transforaminal approach [1,3-5]. A local anesthetic has a temporary pharmacological effect on the spinal cord, while a suspension of corticosteroids, including large particles with possible thromboembolic properties, could cause permanent spinal cord injuries, such as brain or spinal cord infarctions. Although spinal needle position can be confirmed with injections of contrast medium, intravascular contrast medium cannot be observed very well because of the blood flow when part of the contrast medium enters a small vessel. Therefore, in order to minimize the risk of permanent spinal cord injury, some authors have recommended a preliminary injection of local anesthetic before injecting corticosteroids, allowing the operator to assess whether the local anesthetic has entered a blood vessel or not [3,5,9]. Moreover, the use of non-particulate corticosteroids, such as dexamethasone, may also help to minimize the risk of this complication [5,9].

A few authors have reported incidents involving the dissection of an artery, thrombosis formation or massive cerebral edema resulting in deaths that were caused by the perforation of the vertebral artery in a nerve root block performed by the transforaminal route [2,6]. Most operators use aspiration tests and injections of contrast material in order to assess the location of the intravascular needle tip; however, the entry of the needle into an artery may lead to dissection. Although fluoroscopic guidance is generally used during selective nerve root blocks, some authors have recommended CT guidance for clear visualization of not only the needle position but also the surrounding soft tissues and vascular structures to minimize potentially fatal complications. Some authors advocate CT fluoroscopy or digitally subtracted fluoroscopic imaging, which can offer the advantage of real-time visualization of the contrast medium [3,11]. However, there is no consensus of the safest and most effective imaging modalities that should be used for SCNRB.

Guidelines for the cervical transforaminal injection technique involve introducing the needle into the posterior aspect of the intervertebral foramen just anterior to the superior articular process in the oblique view to minimize the risk of injury to the vertebral artery. However, Huntoon [12] reported that more than 20% of cases in which the foramina were dissected had either an ascending or deep cervical artery or a large branch within the needle path of the cervical transforaminal procedure, and one-third consisted of spinal branches that entered the intervertebral foramen posteriorly. Therefore, despite strict adherence to the recommended technique, the cervical transforaminal procedure has a potential risk of unintended puncture of these arteries, leading to serious complications.

## Conclusions

We believe that injecting local anesthetic while confirming the maintenance of the appropriate needle position in the anteroposterior view is important for preventing central needle movement. Because the potential risk of serious complications cannot be completely eliminated during the use of any established SCNRB procedure, preparation for an emergency airway, ventilation and cardiovascular support is indispensable in cases of high spinal cord anesthesia.

## Consent

Written informed consent was obtained from the patient for publication of this case report and any accompanying images. A copy of the written consent is available for review by the Editor-in-Chief of this journal.

## Competing interests

The authors declare that they have no competing interests.

## Authors’ contributions

KT and HK performed the assessment and treatment of the patient. KT was a major contributor in writing the manuscript. SK provided important suggestions regarding medical content. All authors read and approved the final manuscript.

## References

[B1] Brouwers PJ Kottink EJ Simon MA Prevo RL A cervical anterior spinal artery syndrome after diagnostic blockade of the right C6-nerve root Pain 2001 91 397 399 10.1016/S0304-3959(00)00437-1 11275398

[B2] Rozin L Rozin R Koehler SA Shakir A Ladham S Barmada M Dominick J Wecht CH Death during transforaminal epidural steroid nerve root block (C7) due to perforation of the left vertebral artery Am J Forensic Med Pathol 2003 24 351 355 10.1097/01.paf.0000097790.45455.45 14634474

[B3] Baker R Dreyfuss P Mercer S Bogduk N Cervical transforaminal injection of corticosteroids into a radicular artery: a possible mechanism for spinal cord injury Pain 2003 103 211 215 10.1016/S0304-3959(02)00343-3 12749976

[B4] Karasek M Bogduk N Temporary neurologic deficit after cervical transforaminal injection of local anesthetic Pain Med 2004 5 202 205 10.1111/j.1526-4637.2004.04028.x 15209975

[B5] Tiso RL Cutler T Catania JA Whalen K Adverse central nervous system sequelae after selective transforaminal block: the role of corticosteroids Spine J 2004 4 468 474 10.1016/j.spinee.2003.10.007 15246308

[B6] Wallace MA Fukui MB Williams RL Ku A Baghai P Complications of cervical selective nerve root blocks performed with fluoroscopic guidance AJR Am J Roentgenol 2007 188 1218 1221 10.2214/AJR.04.1541 17449763

[B7] Suresh S Berman J Connell DA Cerebellar and brainstem infarction as a complication of CT-guided transforaminal cervical nerve root block Skeletal Radiol 2007 36 449 452 10.1007/s00256-006-0215-0 17216270

[B8] Lubenow T Keh-Wong E Kristof K Ivankovich O Ivankovich AD Inadvertent subdural injection: a complication of an epidural block Anesth Analg 1988 67 175 179 10.1213/00000539-198802001-00175 3341567

[B9] Scanlon GC Moeller-Bertram T Romanowsky SM Wallace MS Cervical transforaminal epidural steroid injections: more dangerous than we think? Spine 2007 32 1249 1256 10.1097/BRS.0b013e318053ec50 17495784

[B10] Furman MB Giovanniello MT O’Brien EM Incidence of intravascular penetration in transforaminal cervical epidural steroid injections Spine 2003 28 21 25 10.1097/00007632-200301010-00007 12544950

[B11] Wagner AL CT fluoroscopic-guided cervical nerve root blocks AJNR Am J Neuroradiol 2005 26 43 44 15661697 PMC7975026

[B12] Huntoon MA Anatomy of the cervical intervertebral foramina: vulnerable arteries and ischemic neurologic injuries after transforaminal epidural injections Pain 2005 117 104 111 10.1016/j.pain.2005.05.030 16055268

